# Diagnostic performance and clinical utility of metagenomic next-generation sequencing in suspected pulmonary infections: a comparative study stratified by immune status

**DOI:** 10.3389/fcimb.2026.1812778

**Published:** 2026-04-22

**Authors:** Tian Li, Jingjing Liu, Xingguang Wang

**Affiliations:** 1 Shandong First Medical University, Jinan, Shandong, China; 2 Shandong Provincial Hospital, Jinan, Shandong, China

**Keywords:** conventional diagnostic methods, immunosuppression, metagenomic next-generation sequencing (mNGS), mixed infection, pathogen spectrum, pulmonary infection

## Abstract

**Background:**

Pulmonary infections represent a significant global health concern, contributing substantially to morbidity and mortality worldwide. Metagenomic next-generation sequencing (mNGS) represents an advanced, comprehensive, and unbiased diagnostic approach for pathogen identification, effectively overcoming many limitations inherent in conventional diagnostic methods. This study aimed to systematically evaluate the clinical performance of mNGS in the etiological diagnosis of pulmonary infections, with a particular emphasis on its utility across diverse immune statuses.

**Methods:**

This retrospective study included 136 patients with suspected pulmonary infections admitted to the Department of Respiratory Medicine at Shandong Provincial Hospital from June 2023 to April 2025. Bronchoalveolar lavage fluid (BALF) samples were collected from all patients and concurrently subjected to mNGS and conventional microbiological testing (CMT). The pathogen detection spectrum and diagnostic performance of mNGS were systematically compared against those of CMT.

**Results:**

mNGS exhibited a significantly higher overall pathogen detection rate compared to CMT (77.2% vs. 50.0%, P < 0.001). Regarding the pathogen spectrum, mNGS identified a broader array of microorganisms, encompassing 19 bacterial, 9 fungal, and 2 mycobacterial species, in contrast to the 11 bacterial, 5 fungal, and 1 mycobacterial species detected by CMT. Diagnostic performance analysis further revealed that mNGS sensitivity was significantly superior to that of CMT (74.6% vs. 46.7%, P < 0.001). Furthermore, mNGS demonstrated a distinct advantage in detecting mixed infections, with a detection rate of 19.1%, significantly exceeding that of CMT (8.8%, P < 0.05). Subgroup analysis indicated a significantly higher incidence of mixed infections in immunocompromised patients compared to immunocompetent patients (35.1% vs. 13.1%, P < 0.05). Additionally, immunocompromised patients were more frequently subjected to adjustments in antimicrobial therapy guided by mNGS results (56.8% vs. 35.4%, χ² = 5.094, P < 0.05).

**Conclusions:**

In conclusion, mNGS offers superior sensitivity and broader pathogen coverage for the etiological diagnosis of pulmonary infections compared to conventional microbiological testing. Its enhanced capability to detect mixed infections significantly improves diagnostic accuracy in immunocompromised patients and effectively facilitates the dynamic optimization of antimicrobial therapy. Serving as a powerful complement to traditional diagnostic methods, mNGS holds particular value for the rapid diagnosis of complex and immunosuppression-associated pulmonary infections.

## 1 Introduction

Pulmonary infections represent a significant global health concern, contributing substantially to morbidity and mortality worldwide. The accurate identification of causative pathogens in lower respiratory tract infections (LRTIs) remains a formidable clinical challenge (Lai et al., 2025). A rapid and precise etiological diagnosis is paramount for guiding targeted antimicrobial therapy, improving patient outcomes, and mitigating antimicrobial overuse. However, conventional microbiological methods are fraught with substantial limitations, including protracted turnaround times (often ranging from several days to weeks), inherently low sensitivity—especially following empirical antibiotic exposure—and restricted detection capabilities for fastidious organisms, anaerobes, and atypical pathogens such as *Pneumocystis jirovecii* (Huang et al., 2025; Li et al., 2024, Li et al., 2025; Xin et al., 2025; Zhuang et al., 2025). These inherent shortcomings frequently result in delayed or ineffective empirical treatment, thereby significantly elevating the mortality risk in critically ill patients (Li et al., 2025).

Metagenomic next-generation sequencing (mNGS) has emerged as an advanced, comprehensive, and unbiased diagnostic tool for pathogen identification in infectious diseases (Huang et al., 2025). As a high-throughput sequencing technology, mNGS operates independently of predefined targets, enabling the simultaneous sequencing of all nucleic acids within a clinical specimen, thus facilitating panoramic detection of potential pathogens (Chen et al., 2025). A growing body of clinical evidence demonstrates that mNGS, particularly when applied to bronchoalveolar lavage fluid (BALF), yields substantially higher pathogen detection rates (70-95%) than conventional microbiological culture methods (12-55%) across a spectrum of pulmonary infections, including community-acquired pneumonia, hospital-acquired pneumonia, and infections in immunocompromised hosts (Deng et al., 2025; Huang et al., 2025; Lai et al., 2025). Significantly, mNGS offers distinct advantages in identifying mixed infections (Huang et al., 2025) and difficult-to-culture or rare pathogens, such as *Chlamydia psittaci* (Li et al., 2025) and non-tuberculous mycobacteria (Xu et al., 2022), thereby effectively bridging critical diagnostic gaps inherent in traditional approaches.

Despite these demonstrable technical strengths, several challenges impede the widespread clinical implementation of mNGS. Firstly, the absence of standardized protocols for sample collection, database construction, and sequencing parameter optimization necessitates context-specific evaluation of diagnostic performance (Zhao L. et al., 2024). Secondly, due to its inherent high sensitivity, mNGS identifies not only bona fide pathogens but also colonizing organisms and background microbiota, rendering accurate clinical interpretation of complex reports a significant challenge for clinicians (Liu Y. et al., 2025). Crucially, the clinical value of mNGS is most pronounced in high-risk populations, particularly immunocompromised patients (Zhao J. et al., 2024, Zhao et al., 2025). These individuals frequently present with more complex pathogen spectra and elevated rates of mixed infections, underscoring an urgent need for precise etiological diagnosis (Qiang et al., 2026). In instances where immunocompromised patients exhibit a suboptimal response to empirical therapy, mNGS can furnish critical etiological evidence to inform targeted antimicrobial adjustments and enhance clinical outcomes (Zhao J. et al., 2024, Zhao et al., 2025).

While prior studies have indicated that mNGS may contribute to a reduction in clinical improvement time for patients with severe pneumonia (Wu et al., 2025), its broader impact on routine clinical practice warrants further comprehensive evaluation. Consequently, the prevailing research paradigm has transitioned from solely technical validation to addressing challenges in clinical interpretation and pinpointing patient populations poised to derive the greatest benefit from this technology. Therefore, this study sought to systematically evaluate the diagnostic performance and clinical utility of mNGS in pulmonary infections, with a specific emphasis on its role across patient populations with varying immune statuses, thereby providing robust evidence-based support for precision diagnosis and treatment.

## 2 Materials and methods

### 2.1 Study design and participants

**Figure 1:**
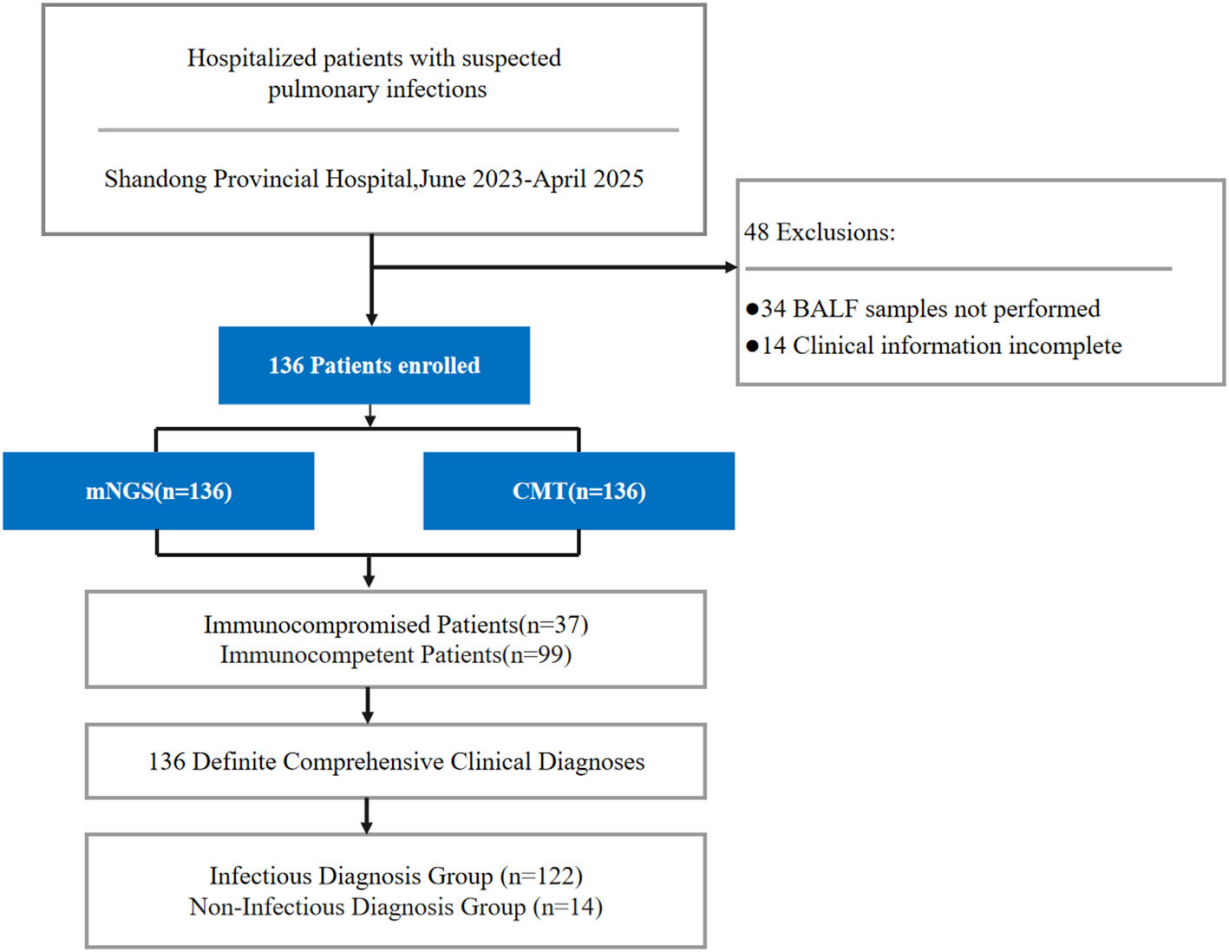
Enrollment details and study design.

A total of 136 hospitalized patients with suspected pulmonary infections, admitted to the Department of Respiratory Medicine at Shandong Provincial Hospital between June 2023 and April 2025, were retrospectively included. Inclusion criteria were defined as follows: (1) age ≥ 18 years; (2) fulfillment of suspected pulmonary infection criteria, characterized by respiratory symptoms or signs (e.g., fever, cough, sputum production, pulmonary rales) accompanied by new or progressive infiltrates on chest imaging; (3) successful completion of bronchoscopy with bronchoalveolar lavage fluid (BALF) sample acquisition meeting laboratory quality requirements; and (4) simultaneous submission of BALF samples for metagenomic next-generation sequencing (mNGS) and conventional microbiological testing (CMT). Exclusion criteria included insufficient clinical records (**Figure 1**). This study was approved by the Ethics Committee of Shandong Provincial Hospital (Approval No. 2026-113), and written informed consent was obtained from all patients or their legal guardians.

### 2.2 Sample collection

BALF samples were collected from all enrolled patients via fiberoptic bronchoscopy following standardized operating procedures established by the Department of Respiratory and Critical Care Medicine. Local anesthesia with 2% lidocaine was administered prior to bronchoscopy. Based on imaging findings, the bronchoscope was advanced into the most affected lung segment or subsegment. Target areas were lavaged multiple times using 20–50 mL of sterile normal saline prewarmed to 37 °C, with a total lavage volume of 100–200 mL and a minimum recovery rate of 30%. At least 40% of the instilled fluid was aspirated and collected into sterile containers. Each BALF sample was aliquoted into two portions and transported under standardized conditions for simultaneous CMT and mNGS analysis (PACEseq, Hugobiotech, Beijing, China).

### 2.3 Detection methods

#### 2.3.1 Conventional microbiological tests

Bacterial pathogens were detected using standard culture techniques. Fungal detection involved smear microscopy with Gram staining, fluorescent staining, and silver staining, as well as antigen-based assays such as β-D-glucan and galactomannan testing. Mycobacterial identification utilized acid-fast staining, smear microscopy, and real-time PCR/nucleic acid amplification testing for rifampicin resistance (Xpert MTB/RIF).

#### 2.3.2 Metagenomic next-generation sequencing and bioinformatic analysis

DNA was extracted from each BALF sample using a nucleic acid extraction kit (YGZZ014, Hugobiotech, Beijing, China). Sequencing libraries were constructed using a universal sequencing library preparation kit (YGZZ005, Hugobiotech). For each batch of experiment, a no-template control (NTC) was set up, taking sterile water as the initial input. Library concentration and integrity were assessed using Qubit 4.0 (Thermo Fisher Scientific, USA) and the Agilent 2100 Bioanalyzer (Agilent Technologies, USA). After quality control, libraries were sequenced on an Illumina 550DX platform with a single-end read length of 75 bp. Raw sequencing data were converted to FASTQ format using bcl2fastq (v2.20.0.422). Adapter sequences, low-quality reads, and reads with high N content were filtered using fastp (v0.24.0), retaining high-quality reads longer than 50 bp. Human-derived sequences were removed by alignment to the human reference genome (GRCh38) using BWA-MEM (v0.7.15). Remaining reads were aligned against a locally curated microbial database constructed from NCBI RefSeq and GenBank databases. Microbial identification was performed based on alignment results, and unique mapped reads and reads per million (RPM) were calculated for each detected microorganism.

Positive mNGS results were defined according to microbial category. For bacteria (excluding *Mycobacterium tuberculosis*), fungi (excluding *Cryptococcus*), and parasites, an mNGS result was considered positive when either of the following criteria was met: (1) the genome coverage of unique reads mapped to the microorganism ranked among the top 10 within the same microbial category and the microorganism was not detected in the negative control (NTC); or (2) the reads per million ratio (RPM-r; RPM_sample_/RPM_NTC)_ was >10 when RPM_NTC_ was not equal to 0. For viruses, *Mycobacterium tuberculosis*, and *Cryptococcus*, an mNGS result was considered positive when the microorganism was not detected in the NTC and at least one unique read was mapped at the species level, or when RPM-r was >5 with RPM_NTC_ ≠ 0.

### 2.4 Definition standards

Immunocompromised status was defined by the presence of any of the following conditions (Kreitmann et al., 2024): (1) active hematologic or solid malignancy; (2) post-solid organ or hematopoietic stem cell transplantation; or (3) long-term use of immunosuppressive agents or systemic corticosteroids. All other patients were classified as immunocompetent.

Mixed infection was defined as the detection of two or more clinically significant pathogenic organisms.

Treatment modifications based on mNGS results were categorized as treatment escalation, treatment de-escalation, or targeted therapy. A composite clinical reference standard, which incorporated the following components: (1) Clinical manifestations: Presence of fever (≥38 °C), localized symptoms (e.g., cough, dyspnea), or systemic symptoms (e.g., fatigue, hypotension);(2) Imaging findings: Chest CT/X-ray showing new or progressive infiltrates, consolidation, or cavitation;(3) Inflammatory markers: elevated levels of C-reactive protein (CRP > 100 mg/L) or procalcitonin (PCT > 0.5 ng/mL);(4) Treatment response: Clinical improvement (e.g., temperature normalization, symptom relief) or radiological resolution after 7 days of targeted antimicrobial therapy.(5) Conventional microbiological results: Positive culture, PCR, or serological results obtained from blood, bronchoalveolar lavage fluid, or other clinically relevant specimens; A case was definitively diagnosed as “pulmonary infection” if it met at least three of the above criteria, including either imaging abnormalities with microbiological confirmation, or imaging abnormalities with a positive treatment response in cases with negative microbiology. Two or more independent clinical experts adjudicated infection status and pathogenic significance, classifying mNGS and CMT results as true positive, false positive, true negative, or false negative.

### 2.5 Statistical analysis

Statistical analyses were performed using SPSS version 27.0 (IBM, USA). Continuous variables were expressed as mean ± standard deviation or median (interquartile range) and compared using Student’s t-test or the Mann-Whitney U test, as appropriate. Categorical variables were expressed as counts (percentages) and compared using the chi-square test or Fisher’s exact test. Diagnostic performance metrics, including sensitivity, specificity, positive predictive value, negative predictive value, and agreement (Kappa coefficient), were calculated using the composite clinical diagnosis as the reference standard. Subgroup analyses were conducted according to immune status. All tests were two-sided, and P < 0.05 was considered statistically significant.

## 3 Results

### 3.1 Baseline characteristics of the patients

A total of 136 patients with suspected pulmonary infections were ultimately enrolled in this study. The detailed baseline characteristics, including age, sex, immune status, and clinical outcomes for each participant, are provided in the **Supplementary Material**. The baseline characteristics of the study population are summarized in **Table 1**. Of these, 81 (59.6%) were male and 55 (40.4%) were female. The median age was 62 years (interquartile range [IQR]: 52–71 years), with an overall range of 22 to 88 years. According to the predefined criteria for immunosuppression, 37 patients (27.2%, 37/136) were classified as immunocompromised. Specifically, this group comprised 15 patients (40.5%) with active hematologic or solid malignancies, 1 patient (2.7%) who had undergone solid organ or hematopoietic stem cell transplantation, and 21 patients (56.8%) receiving long-term immunosuppressive agents or systemic corticosteroids. The remaining 99 patients (72.8%, 99/136) were categorized as immunocompetent. Overall, 73 patients (53.7%) presented with underlying comorbidities, with cerebrovascular diseases (44.1%) and diabetes mellitus (26.9%) being the most prevalent. Histories of smoking and alcohol consumption were present in 30.1% and 26.5% of patients, respectively. No statistically significant differences were observed between the immunocompromised and immunocompetent groups in terms of age, sex, inflammatory markers, or several key baseline comorbidities (all P > 0.05). However, significant differences were noted in the distribution of malignancies and autoimmune diseases between the two groups (P < 0.05). To mitigate potential confounding, multivariable regression analyses (e.g., logistic regression) were subsequently conducted. Following adjustment, disparities in baseline disease distribution did not influence the observed differences in pathogen detection rates between mNGS and CMT across immune status groups, thereby enabling a more accurate assessment of the impact of immune status on diagnostic performance in pulmonary infections.

**Table 1:** Baseline data of the included patients.

Characteristics	Total (n = 136)	Immunocompromised (n = 37)	Immunocompetent (n = 99)	*P* value
Age, years, median (IQR)	62 (52–70.75)	66 (54–72.5)	60 (52–71)	>0.05
Sex, n (%)				>0.05
Male	81 (59.6)	23 (62.2)	58 (58.6)	
Female	55 (40.4)	14 (37.8)	41 (41.4)	
Smoking history, n (%)				>0.05
Yes	41 (30.1)	12 (32.4)	29 (29.3)	
No	95 (69.9)	25 (67.6)	70 (70.7)	
Alcohol consumption, n (%)				>0.05
Yes	36 (26.5)	12 (32.4)	24 (24.2)	
No	100 (73.5)	25 (67.6)	75 (75.8)	
Underlying comorbidities, n (%)				
Cardiovascular and cerebrovascular diseases	41 (44.1)	11 (27.5)	30 (30.3)	>0.05
Diabetes mellitus	25 (26.9)	6 (15.0)	19 (19.2)	>0.05
Renal diseases	2 (2.2)	2 (5.0)	0	>0.05
Autoimmune diseases	3 (3.2)	3 (7.5)	0	<0.05
Malignancy	16 (17.2)	16 (40.0)	0	<0.001
Previous tuberculosis	5 (5.4)	1 (2.5)	4 (7.5)	>0.05
Hematological diseases	1 (1.1)	1 (2.5)	0	>0.05

### 3.2 Overall pathogen detection rates of mNGS and CMT

Utilizing BALF samples, mNGS identified clinically significant pathogen-derived nucleic acid sequences in 105 patients, resulting in an overall positivity rate of 77.2% (105/136). In contrast, the overall positivity rate of conventional microbiological testing was 50.0% (68/136). The pathogen detection rate of mNGS was significantly higher than that of CMT (P < 0.001) (**Table 2**).

**Table 2:** Comparison of pathogen detection rates between mNGS and CMT in BALF samples.

Sample type	Testing method	Positive cases	Positive rate (%)	P value
BALF(n=136)	mNGS	105	77.2	<0.001
	CMT	68	50.0	

### 3.3 Concordance and diagnostic performance of mNGS and CMT

**Figure 2:**
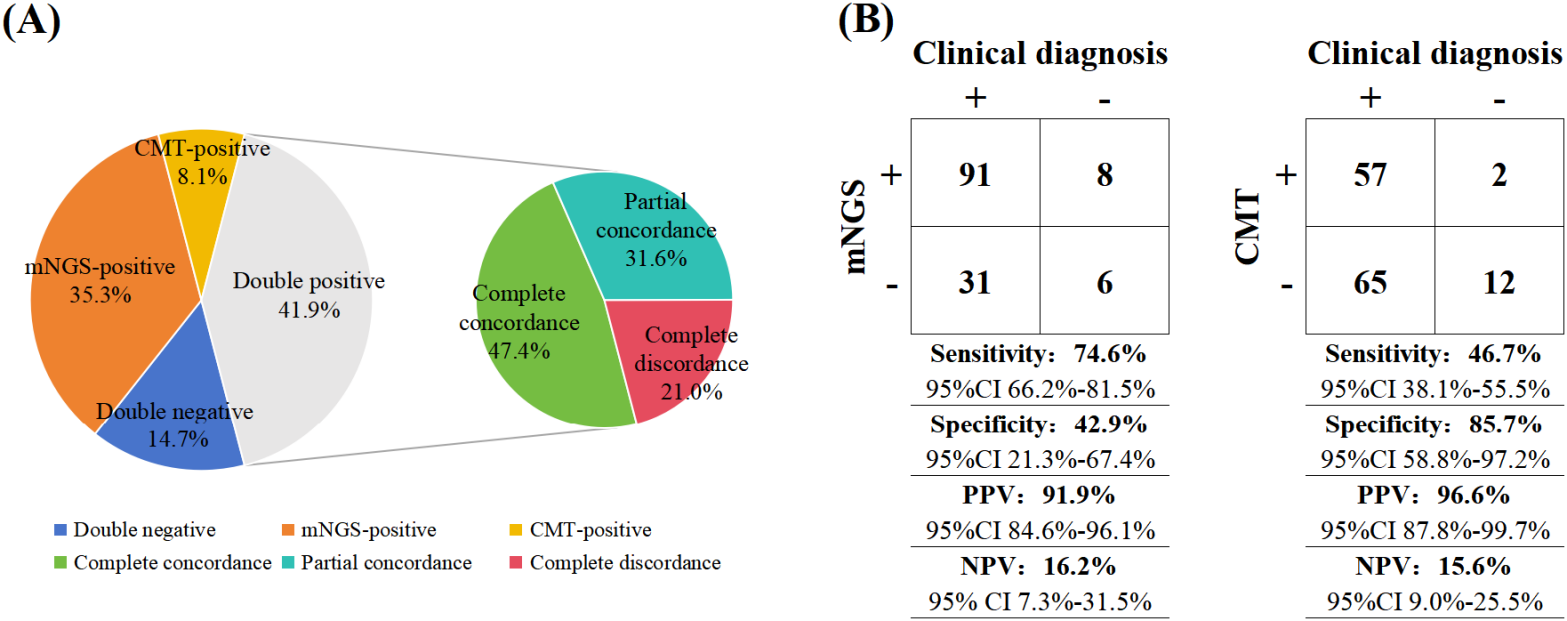
Concordance between mNGS and CMT **(A)**. Diagnostic performance of mNGS and CMT in pulmonary infections, including Sensitivity, Specificity, PPV, and NPV for Each Test **(B)**. The ‘PPV’ is Positive Predictive Value, ‘NPV’ is Negative Predictive Value, and ‘CI’ represents Confidence Interval.

Among the 136 patients, the overall concordance rate between mNGS and CMT for pathogen detection was 56.6% (77/136). Specifically, 57 patients (41.9%) tested positive by both methods, while 20 patients (14.7%) tested negative by both. Among the cases positive by both methods, complete pathogen concordance was observed in 27 patients, partial concordance in 18 patients, and complete discordance in 12 patients. Additionally, 48 patients (35.3%) were positive solely by mNGS, while 11 patients (8.1%) were positive solely by CMT (**Figure 2**). We performed a detailed analysis of the “mNGS-positive only” subgroup. Among these, 12 patients (25.0%) were immunocompromised. The pathogen spectrum was predominantly composed of opportunistic pathogens (e.g., *Pseudomonas aeruginosa*, *Acinetobacter baumannii*), accounting for 16 cases (33.3%), followed by fungi (e.g., *Aspergillus*, *Mucorales*, *Pneumocystis jirovecii*) in 10 cases (20.8%). Pathogens with strict clinical significance (e.g., *Mycobacterium tuberculosis*, *Mycobacterium intracellulare*) were detected in 3 cases (6.3%).Based on a comprehensive analysis of host immune status and inflammatory markers (white blood cell count [WBC], PCT, CRP), these cases were classified into two categories: (1) Highly suspected clinical infection (n=30, 62.5%): these patients were predominantly immunocompromised (10/12, 83.3%) and often presented with significantly elevated levels of WBC, PCT, or CRP at the time of sampling. (2) Likely colonization or contamination (n=18, 37.5%): this category was more common among immunocompetent patients, who lacked corresponding clinical symptoms or abnormalities in inflammatory markers. Retrospective analysis revealed an overall clinical adoption rate of 37.5% (18/48) for the “mNGS-positive only” results. The treatment adjustment rate for cases adjudicated as “highly suspected clinical infection” was 56.7% (17/30), which was significantly higher than the 5.6% (1/18) observed for cases deemed “likely colonization or contamination” (p < 0.001). The odds ratio (OR) for treatment adjustment between the two groups was 22.6 (95% CI: 2.7–188.1).

Kappa analysis demonstrated poor agreement between the two methods for pathogen detection (Kappa = 0.132). With the composite clinical diagnosis serving as the reference standard, 122 patients (89.7%) were ultimately diagnosed with pulmonary infection, whereas 14 patients were determined to have non-infectious pulmonary or extrapulmonary diseases. The sensitivity of mNGS (74.6%, 95% confidence interval [CI]: 66.2%-81.5%) was significantly higher than that of CMT (46.7%, 95% CI: 38.1%-55.5%) (P < 0.001). Conversely, the specificity of CMT (85.7%, 95% CI: 58.8%–97.2%) was significantly higher than that of mNGS (42.9%, 95% CI: 21.3%-67.4%) (P < 0.05). The positive predictive value (PPV) and negative predictive value (NPV) of mNGS were 91.9% (95% CI: 84.6%-96.1%) and 16.2% (95% CI: 7.3%-31.5%), respectively, while those of CMT were 96.6% (95% CI: 87.8%-99.7%) and 15.6% (95% CI: 9.0%–25.5%). No statistically significant differences were observed between the two methods in terms of PPV or NPV (P > 0.05) (**Figure 2**). Regarding turnaround time, the median reporting time for mNGS was 1 day (IQR: 1-1), which was significantly shorter than that for CMT (6.8 days; IQR: 2-7) (U = 18,496.000, P < 0.001).

### 3.4 Comparison of pathogen detection rates between mNGS and CMT across different immune statuses

**Figure 3:**
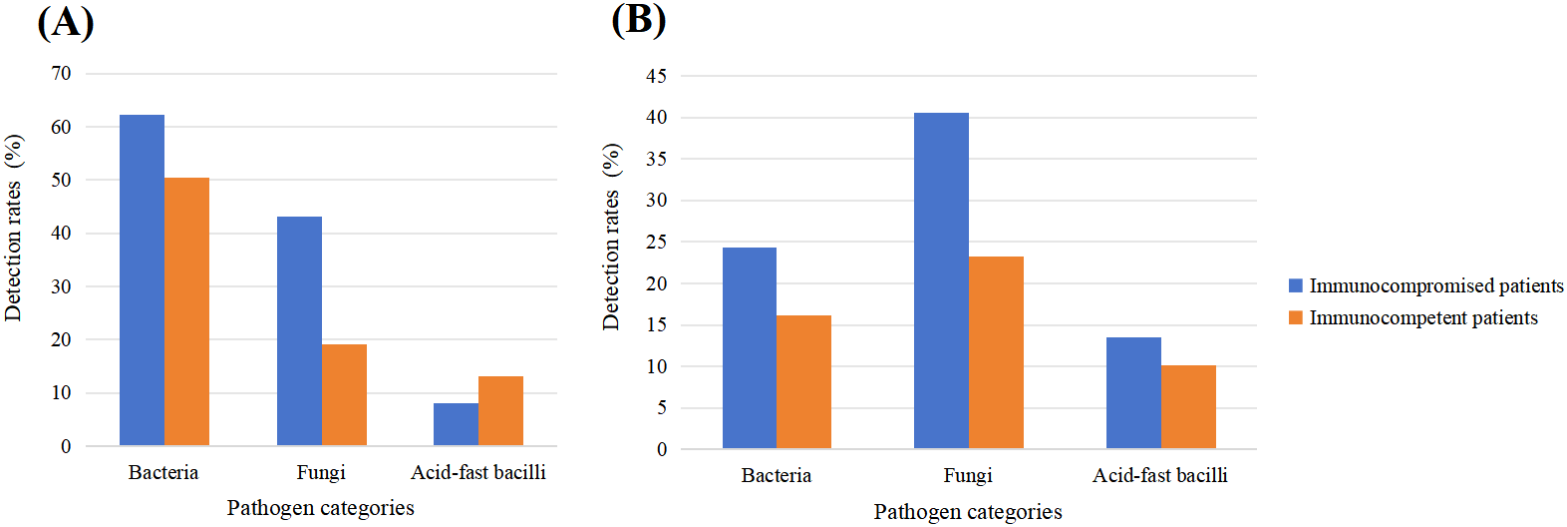
Pathogen detection rates by mNGS in immunocompromised and immunocompetent patients **(A)**. Pathogen detection rates by CMT in immunocompromised and immunocompetent patients **(B)**.

In immunocompromised patients, pathogen detection rates varied between mNGS and CMT across different pathogen categories. For bacterial pathogens, the detection rates of mNGS was 62.2% (23/37), which was significantly higher than that of CMT (24.3%, 9/37) (P < 0.05). For fungal pathogens, the detection rates of mNGS and CMT were 43.2% (16/37) and 40.5% (15/37), respectively, with no statistically significant difference according to McNemar’s exact test (P > 0.05). For acid-fast bacilli, detection rates were 8.1% (3/37) for mNGS and 13.5% (5/37) for CMT, with no significant difference observed (P > 0.05) (**Figure 3**).

A similar overall pattern was observed among immunocompetent patients. mNGS maintained a significant advantage in bacterial detection, with a positivity rate of 50.5% (50/99), compared with 16.2% (16/99) for CMT (P < 0.001). For fungal pathogens, detection rates were 19.2% (19/99) for mNGS and 23.2% (23/99) for CMT, with no significant difference (P > 0.05). For acid-fast bacilli, mNGS showed a slightly higher detection rate than CMT (13.1% vs. 10.1%), although the difference was not statistically significant (P > 0.05) (**Figure 3**).

Further comparison between immune status groups using the same diagnostic method (**Figure 3**) revealed that the fungal detection rate by mNGS was significantly higher in immunocompromised patients than in immunocompetent patients (43.2% vs. 19.2%, P < 0.05). In contrast, no significant difference in fungal detection was observed between the two groups when using CMT (40.5% vs. 23.2%, P > 0.05). For bacterial and acid-fast bacilli detection, no significant differences were identified between immune status groups using either method (P > 0.05).

### 3.5 Pathogen spectrum characteristics

Parallel testing with mNGS and CMT provided a comprehensive characterization of the pathogen spectrum in patients with pulmonary infections. A total of 32 distinct pathogens were identified across all 136 samples, including 21 bacterial species, 9 fungal species, and 2 mycobacterial categories (Mycobacterium tuberculosis complex and non-tuberculous mycobacteria). mNGS detected 30 pathogens (19 bacteria, 9 fungi, and 2 mycobacteria) (**Table 3**), whereas CMT identified only 17 pathogens (11 bacteria, 5 fungi, and 1 mycobacterium) (**Table 4**). Among bacterial pathogens detected by mNGS, *Pseudomonas aeruginosa* was the most prevalent (n = 29), followed by *Haemophilus parainfluenzae* (n = 11) and *Klebsiella pneumoniae* (n = 8). Notably, nine bacterial species including *Nocardia farcinica*, *Haemophilus influenzae*, *Legionella pneumophila*, *Corynebacterium striatum*, *Proteus mirabilis*, *Enterococcus faecalis*, *Moraxella catarrhalis*, *Nocardia abscessus*, and *Nocardia otitidiscaviarum* were detected exclusively by mNGS (**Table 5**).

**Table 3:** Pathogen categories and species identified by mNGS.

Pathogen category	Pathogen species
Bacteria	*Klebsiella pneumoniae*; *Pseudomonas aeruginosa*; *Nocardia farcinica*; *Haemophilus influenzae*; *Legionella pneumophila*; *Enterococcus faecalis*; *Corynebacterium striatum*; *Staphylococcus aureus*; *Proteus mirabilis*; *Achromobacter xylosoxidans*; *Enterococcus faecium*; *Escherichia coli*; *Moraxella catarrhalis*; *Nocardia abscessus*; *Enterobacter cloacae* complex; *Acinetobacter baumannii*; *Streptococcus pneumoniae*; *Nocardia otitidiscaviarum*; *Serratia marcescens*
Fungi	*Candida albicans*; *Aspergillus flavus*; *Aspergillus niger*; *Rhizopus delemar*; *Mucor racemosus*; *Rhizopus microsporus*; *Aspergillus tubingensis*; *Aspergillus fumigatus*; *Pneumocystis jirovecii*
Mycobacteria	*Mycobacterium tuberculosis* complex; *Mycobacterium intracellulare*

**Table 4:** Pathogen categories and species identified by conventional microbiological tests.

Pathogen category	Pathogen species
Bacteria	*Escherichia coli*; *Pseudomonas aeruginosa*; *Achromobacter xylosoxidans* subsp. *xylosoxidans*; *Enterococcus avium*; *Klebsiella pneumoniae*; *Enterococcus faecalis*; *Enterobacter cloacae*; *Staphylococcus aureus*; *Acinetobacter baumannii*; *Serratia marcescens*; *Streptococcus pneumoniae*
Fungi	*Candida albicans*; *Candida krusei*; *Aspergillus fumigatus*; *Aspergillus flavus*; *Aspergillus niger*
Mycobacteria	*Mycobacterium tuberculosis* complex

**Table 5:** Pathogens exclusively detected by mNGS.

Pathogen category	Pathogen species
Bacteria	*Escherichia coli*; *Pseudomonas aeruginosa*; *Achromobacter xylosoxidans* subsp. *xylosoxidans*; *Enterococcus avium*; *Klebsiella pneumoniae*; *Enterococcus faecalis*; *Enterobacter cloacae*; *Staphylococcus aureus*; *Acinetobacter baumannii*; *Serratia marcescens*; *Streptococcus pneumoniae*
Fungi	*Candida albicans*; *Candida krusei*; *Aspergillus fumigatus*; *Aspergillus flavus*; *Aspergillus niger*
Mycobacteria	*Mycobacterium tuberculosis* complex

The overall bacterial detection rate of mNGS (53.7%, 73/136) was significantly higher than that of CMT (18.4%, 25/136) (χ² = 54.98, P < 0.001). For fungal pathogens, *Aspergillus fumigatus* (n = 17) was the most frequently detected species by both methods. Importantly, several clinically relevant fungi, including *Pneumocystis jirovecii* and members of the order Mucorales (e.g., *Mucor racemosus*, *Rhizopus microsporus*, and *Rhizomucor pusillus*), were detected exclusively by mNGS. The fungal detection rate of mNGS was 25.7% (35/136), which was no statistically significantly different from that of CMT (27.9%, 38/136) (χ² = 0.059, P > 0.05). Similarly, the detection rate of acid-fast bacilli was 11.8% (16/136) for mNGS and 11.0% (15/136) for CMT, also demonstrating a no statistically significant difference (P > 0.05).

### 3.6 Performance of mNGS and CMT in the diagnosis of mixed infections

**Figure 4:**
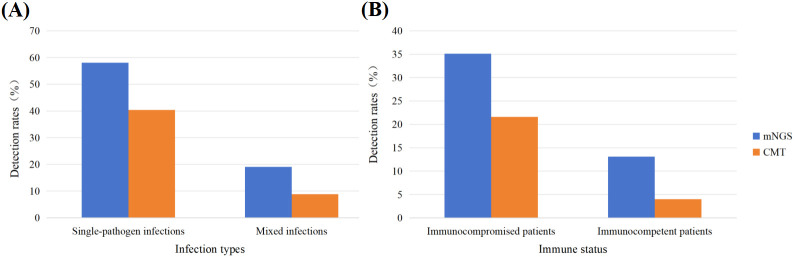
Comparison of single and mixed pathogen infections detected **(A)**. Comparison of the detection rates of mixed infections between Immunocompromised and Immunocompetent patients **(B)**.

Among the 105 mNGS-positive cases, 79 patients (58.1%, 79/136) presented with single-pathogen infections, whereas 26 patients (19.1%, 26/136) were diagnosed with mixed infections (**Figure 4**). In contrast, CMT identified 67 positive cases, including 55 single infections (40.4%, 55/136) and 12 mixed infections (8.8%, 12/136). Overall, the detection rate of mixed infections was significantly higher with mNGS than with CMT (19.1% vs. 8.8%, P < 0.05). Further stratification by infection type revealed that bacterial infections predominated among single-pathogen infections. In mixed infections, mNGS revealed a broader spectrum of pathogen combinations. The most common mixed infection pattern was bacterial–fungal co-infection, identified in 15 cases (11.0%, 15/136), followed by bacterial–bacterial co-infection in 5 cases (3.7%, 5/136). CMT showed limited ability to detect these major mixed infection patterns, identifying only 8 bacterial–fungal infections (5.9%, 8/136) and 1 bacterial–bacterial infection (0.7%, 1/136). Stratified analysis by immune status demonstrated that the detection rate of mixed infections by mNGS was significantly higher in immunocompromised patients than in immunocompetent patients (35.1% [13/37] vs. 13.1% [13/99], P < 0.05). Similarly, under CMT, the mixed infection detection rate was also higher in immunocompromised patients (21.6% [8/37] vs. 4.0% (Huang et al., 2025)/99], P < 0.05) (**Figure 4**).

### 3.7 Impact of mNGS on treatment modification across different immune statuses

**Figure 5:**
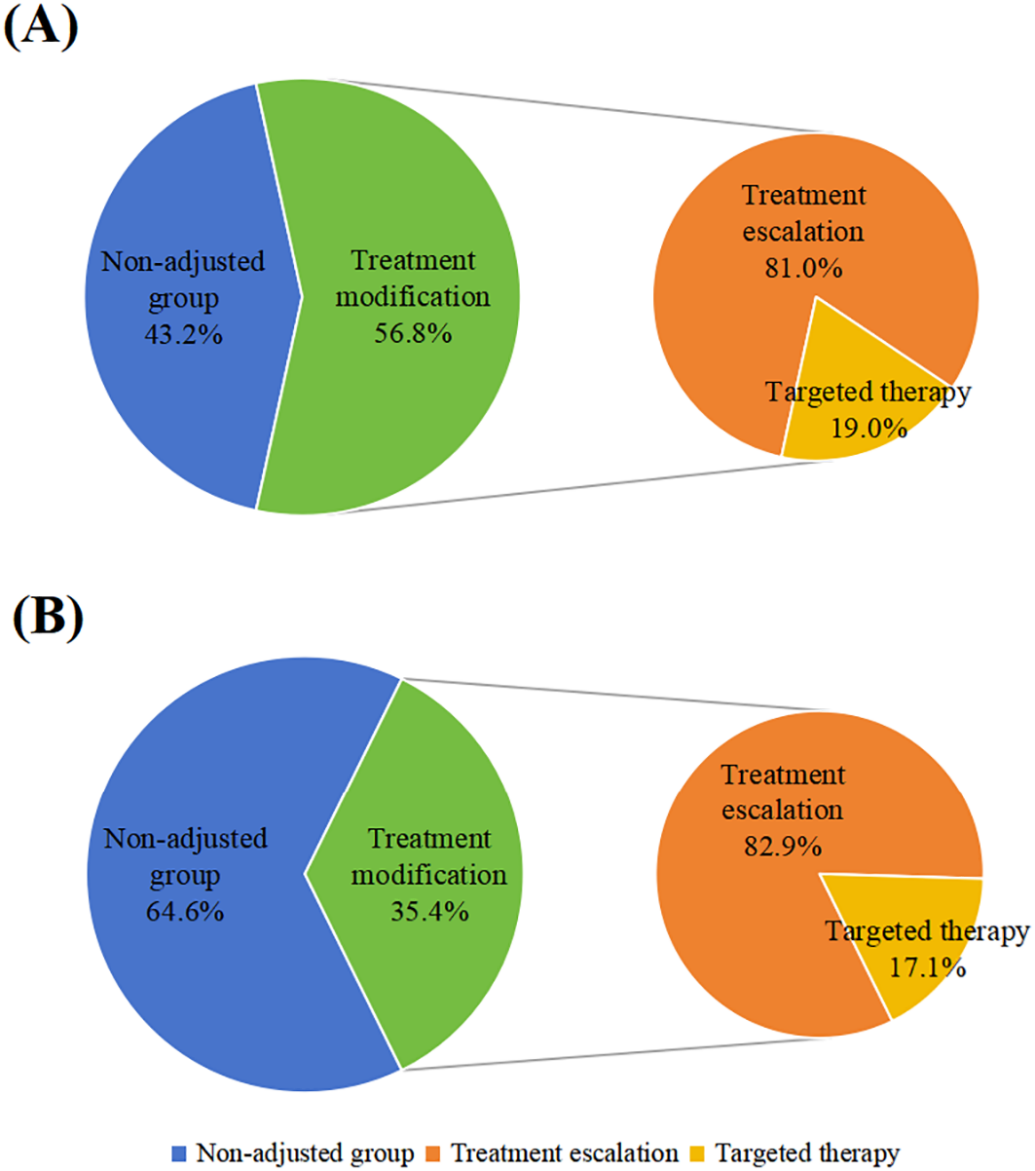
Proportion of treatment modification in immunocompromised patients **(A)**. Proportion of treatment modification in Immunocompetent patients **(B)**.

The clinical utility of mNGS was further evaluated by assessing the modification of treatment regimens based on mNGS results. Immunocompromised patients demonstrated a significantly higher propensity to rely on mNGS findings for guiding therapeutic adjustments. In the immunocompromised group, 56.8% (21/37) of patients underwent treatment modification based on mNGS results, a proportion significantly higher than that observed in the immunocompetent group (35.4%, 35/99) (χ² = 5.094, P < 0.05). Regarding adjustment strategies, both groups exhibited a high degree of consistency, with treatment escalation being the predominant approach. The proportions of treatment escalation were 81.0% (17/21) in the immunocompromised group and 82.9% (29/35) in the immunocompetent group, with no statistically significant difference (P > 0.05). The remaining patients received targeted therapy (19.0% vs. 17.1%), with no instances of treatment de-escalation observed (**Figure 5**).

## 4 Discussion

Rapid and accurate etiological diagnosis is a cornerstone for improving clinical outcomes in patients with pulmonary infections and for optimizing antimicrobial therapy. Previous studies have consistently demonstrated that inappropriate or delayed initial empirical treatment is associated with a significantly increased risk of mortality (GBD 2019 LRI Collaborators, 2022; Li et al., 2025). However, the widespread use of empirical antibiotics (Ito et al., 2024), the slow growth of many pathogens in culture, and the stringent requirements for culture conditions (Demir et al., 2025) substantially compromise the sensitivity of CMT. mNGS as a culture-independent and unbiased high-throughput technology, theoretically overcomes several inherent limitations of traditional diagnostic approaches and offers a promising tool for pathogen identification in infectious diseases.

In this retrospective study of 136 patients with suspected pulmonary infection, mNGS based on BALF demonstrated a significantly higher overall pathogen detection rate than CMT, a finding consistent with multiple prior investigations (Chen et al., 2025; Huang et al., 2025). Notably, mNGS identified a broader pathogen spectrum, detecting 30 unique pathogens compared with 17 identified by CMT, underscoring its superior breadth in identifying etiological agents (Huang et al., 2025). When stratified by pathogen type, mNGS showed a marked advantage in bacterial detection, with a detection rate of 53.7% compared with 18.4% for CMT (P < 0.001). This finding aligns with the results reported by Deng et al., who demonstrated that metagenomic sequencing substantially outperforms conventional methods in bacterial pathogen detection (Deng et al., 2025). In contrast, for fungal and mycobacterial infections, although the overall detection rates of mNGS and CMT appeared numerically comparable, no statistically significant disparities were noted, Despite the lack of overall statistical difference, mNGS confers distinct advantages in identifying specific fungal pathogens. In particular, mNGS exhibited markedly higher sensitivity in detecting *Pneumocystis jirovecii* and *Mucorales* fungi, which are notoriously difficult to culture. These findings corroborate previous reports demonstrating the superior performance of mNGS in fungal diagnostics (Chen et al., 2025; Li et al., 2023; Wang et al., 2022). It is noteworthy, however, that Shi et al. reported higher sensitivity for *Aspergillus* detection using a combination of culture and galactomannan assays compared with mNGS (100% vs. 66.7%, P = 0.033) (Shi et al., 2022), which may partially explain the lack of a clear overall advantage of mNGS for all fungal infections in this cohort.

For mycobacterial infections, including Mycobacterium tuberculosis and non-tuberculous mycobacteria (NTM), conventional culture is limited by prolonged turnaround time, while smear microscopy suffers from low sensitivity. By directly detecting pathogen nucleic acids, mNGS has the potential to provide earlier diagnostic clues (Xu et al., 2022). Although similar detection rates for mycobacteria were observed between mNGS and CMT in this study, mNGS may still offer clinical advantages by enabling earlier diagnosis and facilitating the identification of mixed NTM infections or resistance-associated genetic markers (Zhang et al., 2023; Liu B. et al., 2025). The concordance analysis revealed a low level of agreement between mNGS and CMT (Kappa = 0.132), consistent with previous studies (Chen et al., 2025). This low concordance primarily reflects the broader detection spectrum of mNGS, as more than one-third of patients (35.3%) were positive only by mNGS. This phenomenon was particularly evident in immunocompromised patients and in cases of mixed infections, where mNGS identified pathogens beyond the scope of routine testing (Zhao et al., 2025; Qiang et al., 2026). Conversely, the high sensitivity of mNGS may introduce challenges related to distinguishing true pathogens from colonizing or contaminating organisms (Liu Y. et al., 2025). Importantly, the low statistical concordance does not indicate a limitation of mNGS itself but rather underscores the complementary nature of mNGS and CMT in clinical practice.

From a clinical perspective, mNGS serves as a rapid and broad-spectrum screening tool, particularly valuable for critically ill patients, complex cases, immunocompromised hosts, or patients with negative conventional test results, in whom rapid narrowing of the etiological spectrum is crucial (Baillie et al., 2023; Feng et al., 2024; Alcolea-Medina et al., 2025). Conversely, CMT, including culture and antimicrobial susceptibility testing, remains indispensable for confirming pathogen viability and guiding precise antimicrobial selection (Gan et al., 2024). Accumulating evidence suggests that the combined use of mNGS and CMT can significantly enhance pathogen detection rates, optimize antimicrobial regimens, shorten time to clinical improvement, and reduce mortality (Wu et al., 2025; Xu et al., 2025; Zhu et al., 2025), highlighting their complementary roles across different diagnostic dimensions (Chen et al., 2025).

In terms of diagnostic performance, mNGS demonstrated significantly higher sensitivity than CMT (74.6% vs. 46.7%), consistent with prior reports (Mu et al., 2021; Zhuang et al., 2025). However, CMT showed superior specificity compared with mNGS (85.7% vs. 42.9%). This discrepancy is not a result of a technical flaw but stems from fundamental differences in the detection principles of the two methods. mNGS employs an unbiased sequencing strategy that enables high-throughput sequencing of all nucleic acids present in clinical samples, including those from nonviable microorganisms, reagent-derived background bacteria, environmental contaminants, and human commensal microbiota (Miller and Chiu, 2022). In contrast, CMT relies on the ability of microorganisms to actively grow or on targeted recognition mechanisms for specific pathogens, which inherently filter out clinically insignificant signals, such as those from nonviable microorganisms, contaminants, or commensal microbiota (Chen J. et al., 2024). The high sensitivity of mNGS is often accompanied by the detection of colonizing organisms, background nucleic acids, or clinically ambiguous microorganisms, particularly in lower respiratory tract specimens where microbial communities are complex and commensal flora are abundant. False-positive or ambiguous signals in mNGS are often due to contamination during the experimental process (e.g., environmental or reagent-derived background microorganisms) (Diao et al., 2023), as well as interference from normal commensal flora and microbiota (Diao et al., 2022). In this study, by integrating host immune status, inflammatory markers, imaging findings, sequence read counts and treatment response (Fourgeaud et al., 2024; Liu Y. et al., 2025), an in-depth analysis was conducted on the “mNGS-positive only” subgroup. The results indicate that host immune status is a critical variable for distinguishing clinical infection from colonization/contamination. In immunocompromised patients, the detection of *Pneumocystis jirovecii* or *Aspergillus* species, even at low sequencing read counts, has a high positive predictive value (Sprute et al., 2023); this population constituted the majority of the “highly suspected clinical infection” subgroup and was often accompanied by significantly elevated inflammatory markers. In contrast, the detection of opportunistic pathogens (such as *Pseudomonas aeruginosa* or *Acinetobacter baumannii*) in immunocompetent patients is more likely to be interpreted as colonization or contamination (Reynolds and Kollef, 2021). Additionally, the significantly shorter turnaround time of mNGS observed in this study (P < 0.001) represents a critical clinical advantage, enabling earlier therapeutic decision-making, as also demonstrated in previous studies (Charalampous et al., 2024; Tan et al., 2024). mNGS also significantly outperformed CMT in the detection of mixed infections (19.1% vs. 8.8%). Mixed infections are frequently underdiagnosed using traditional methods, particularly in immunocompromised patients (Huang et al., 2025; Xin et al., 2025; Zhao et al., 2025). In this cohort, bacterial–fungal co-infection was the most common mixed infection pattern, accounting for 11.0% of cases, whereas CMT showed limited sensitivity for such combinations. These findings are consistent with prior studies demonstrating the superior ability of mNGS to detect bacterial–fungal co-infections, including combinations involving *Pneumocystis jirovecii* and *Aspergillu*s species (Miller et al., 2020; Li et al., 2021).

Further stratified analysis revealed that immune status substantially influenced both pathogen detection rates and the clinical utility of mNGS. Immunocompromised patients exhibited significantly higher fungal detection rates (43.2% vs. 19.2%) and mixed infection rates (35.1% vs. 13.1%) compared with immunocompetent patients. Moreover, treatment modifications guided by mNGS were more frequent in the immunocompromised group (56.8% vs. 35.4%). These findings align with the well-established increased susceptibility of immunocompromised patients to opportunistic fungal infections (Elalouf et al., 2023; Bongomin et al., 2024). Consistent with the study by Lin et al. (2022), our results indicate that mNGS provides substantial incremental diagnostic value in immunocompromised populations by revealing complex pathogen spectra, including rare and mixed infections. Early application of BALF-mNGS in this high-risk population may therefore facilitate more precise antimicrobial adjustments and ultimately improve clinical outcomes (Zhao J. et al., 2024). Furthermore, our study demonstrated that immunocompromised patients were significantly more likely than immunocompetent patients to undergo anti-infective treatment modifications based on BALF-mNGS results. This finding highlights the critical role of mNGS in guiding therapeutic decision-making for complex pulmonary infections. Consistent with previous reports, including the study by Chen et al. in HIV-infected patients with *Pneumocystis jiroveci*i pneumonia (Chen Q. et al., 2024), mNGS frequently identified additional pathogens and prompted treatment adjustments. Importantly, escalation of antimicrobial therapy constituted the predominant adjustment strategy in both immune groups, underscoring the central clinical value of mNGS in uncovering pathogens not covered by empirical regimens, a benefit that is particularly pronounced in immunocompromised patients with complex infectious profiles.

This study has several limitations. First, its retrospective design may have introduced selection bias. Because the analysis was based on retrospectively collected clinical data, the completeness and standardization of medical records may have affected the representativeness of the pathogen detection results. In addition, the exclusion of patients with incomplete testing data or loss to follow-up may have influenced the evaluation of the diagnostic performance of mNGS compared with conventional methods. Second, this was a single-center study, which may limit the generalizability of the findings. As the patient population, regional epidemiological characteristics, distribution of immune status, and clinical management model were specific to this setting, caution is needed when extrapolating these conclusions to other regions or institutions. Third, although this study assessed the pathogen detection rate and diagnostic efficacy of mNGS, it did not directly correlate test results with clinical outcomes, such as 28-day mortality, length of hospital stay, or medical costs. Therefore, the actual clinical benefit of this technology could not be fully evaluated. Fourth, mNGS itself has inherent limitations. One major challenge is the lack of universally accepted and standardized clinical interpretation thresholds. The determination of a positive result still largely depends on the clinician’s comprehensive judgment based on the patient’s clinical manifestations, imaging findings, and immune status, which may increase the risk of subjective bias. In addition, differences in technical details among mNGS platforms, including DNA/RNA library preparation, host depletion efficiency, and database coverage, may lead to heterogeneity in detection results. Therefore, the conclusions derived from the single technical protocol used in this study may not fully reflect the performance of other platforms. Future studies should address these limitations through prospective, multicenter cohort designs, incorporate well-defined clinical outcome indicators such as 28-day mortality and ICU length of stay, and include cost-effectiveness analyses to better evaluate the real-world clinical value of mNGS.

## 5 Conclusion

In summary, this study demonstrates that bronchoalveolar lavage fluid-based metagenomic next-generation sequencing offers higher sensitivity, broader pathogen coverage, and a shorter turnaround time than conventional microbiological testing for the etiological diagnosis of pulmonary infections. mNGS shows particular advantages in detecting mixed infections, difficult-to-culture organisms, and rare pathogens, and provides unique diagnostic value in immunocompromised patients. Nevertheless, the relatively low specificity of mNGS limits its ability to fully replace traditional culture-based methods. Therefore, an integrated diagnostic strategy that combines the high sensitivity of mNGS with the high specificity of conventional culture, while incorporating clinical context and host immune status, represents a promising direction for improving the accuracy and effectiveness of pulmonary infection diagnosis in future clinical practice.

## Data Availability

The datasets generated and analyzed during the current study are not publicly available due to 552 privacy restrictions. Requests to access the datasets should be directed to xingguang-wang@163.com.
